# Unravelling the Immunomodulatory Effects of Viral Ion Channels, towards the Treatment of Disease

**DOI:** 10.3390/v13112165

**Published:** 2021-10-27

**Authors:** Siobhan Gargan, Nigel J. Stevenson

**Affiliations:** 1 Viral Immunology Group, School of Biochemistry and Immunology, Trinity Biomedical Sciences Institute, Trinity College Dublin, D02 R590 Dublin, Ireland; GARGANSI@tcd.ie; 2 Viral Immunology Group, Royal College of Surgeons in Ireland-Medical University of Bahrain, Manama 15503, Bahrain

**Keywords:** viruses, ion channels, viroporins, immune evasion, immune modulation, influenza, HIV, HCV, SARS-CoV, HPV

## Abstract

The current COVID-19 pandemic has highlighted the need for the research community to develop a better understanding of viruses, in particular their modes of infection and replicative lifecycles, to aid in the development of novel vaccines and much needed anti-viral therapeutics. Several viruses express proteins capable of forming pores in host cellular membranes, termed “Viroporins”. They are a family of small hydrophobic proteins, with at least one amphipathic domain, which characteristically form oligomeric structures with central hydrophilic domains. Consequently, they can facilitate the transport of ions through the hydrophilic core. Viroporins localise to host membranes such as the endoplasmic reticulum and regulate ion homeostasis creating a favourable environment for viral infection. Viroporins also contribute to viral immune evasion via several mechanisms. Given that viroporins are often essential for virion assembly and egress, and as their structural features tend to be evolutionarily conserved, they are attractive targets for anti-viral therapeutics. This review discusses the current knowledge of several viroporins, namely Influenza A virus (IAV) M2, Human Immunodeficiency Virus (HIV)-1 Viral protein U (Vpu), Hepatitis C Virus (HCV) p7, Human Papillomavirus (HPV)-16 E5, Severe Acute Respiratory Syndrome Coronavirus (SARS-CoV) Open Reading Frame (ORF)3a and Polyomavirus agnoprotein. We highlight the intricate but broad immunomodulatory effects of these viroporins and discuss the current antiviral therapies that target them; continually highlighting the need for future investigations to focus on novel therapeutics in the treatment of existing and future emergent viruses.

## 1. Introduction

Abbreviations: TMDs, transmembrane domain; MHC, Major Histocompatibility Complex.

**Figure 1:**
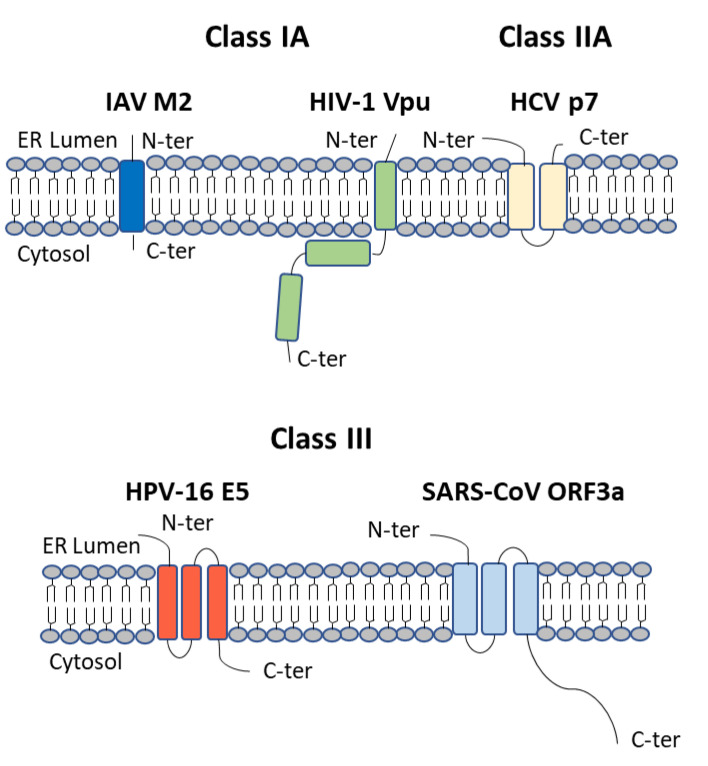
Viroporin Structure. Viroporins localise to intracellular membranes and possess at least one membrane spanning domain. They are divided into classes depending on the number of trans-membrane domains (TMDs). IAV M2 is a Class IA viroporin with a single TMD, a luminal N-terminus and C-terminal cytoplasmic tail. Vpu, also a class IA viroporin, is comprised of a short N-terminal domain, a hydrophobic TMD and a longer cytoplasmic domain, which includes the hinge region and two α-helical domains. HCV p7 is a class IIA viroporin with two transmembrane domains and luminal N and C termini. HPV-16 E5 and SARS-Co-V ORF3a are both class III viroporins with three membrane-spanning domains while SARS-CoV ORF3a has a long cytoplasmic tail.

Viroporins were first discovered due the ability of viruses to modify host plasma membrane permeability [1]. Members of the viroporin family are all small hydrophobic proteins, typically 60–120 amino acids in length, which oligomerise following their insertion into host cellular membranes [2]. Their amphipathic α-helical domains allow for homo-oligomerisation, which results in the formation of an aqueous pore in the membrane. The hydrophilic core of the ion channel then allows for the transport of ions or other small molecules across the membrane, modifying ion homeostasis and cellular processes. Viroporins can be further divided into class I, II, and III, depending on the number of transmembrane domains (TMDs) [3]. Class I viroporins, with a single TMD, are further subdivided into Class IA with a luminal N-terminus and C-terminal cytoplasmic tail and Class IB with the opposing topology [4]. Class II viroporins have two TMDs joined by a basic loop and are further divided into Class IIA with a luminal N and C-terminus and Class IIB with cytosolic termini [4] (Figure 1).

Viroporins are involved in numerous processes during the viral life cycle, but their main function is to regulate virion assembly and egress from the infected cell [4]. The dysregulation of ions can also trigger the activation of inflammasomes, leading to autophagy and apoptosis [5]. The release of Ca^2+^ ions from the mitochondria and Golgi, or the influx of extracellular Ca^2+^ ions, can aid virion production and release and can also lead to apoptosis of infected cells [6]. Furthermore, many viroporins also have functions independent of their channel forming ability, including immune evasion mechanisms, such as the downregulation of Major Histocompatibility Complex (MHC) molecules from the cell surface to avoid immune detection [7,8,9]. Viroporins are essential for the release of many viruses making them a potential therapeutic target for the treatment of infectious viruses [4]. Indeed, compounds which block their pore forming capabilities, such as rimantadine, are being used in current treatment strategies [10].

## 2. IAV M2

Abbreviations: IAV, Influenza A virus; IBV, Influenza B virus; HA, Haemagglutinin; NA, Neuraminidase; NS, non-structural protein; M2; Matrix protein 2; IFN, interferon; vRNP, viral ribonucleoprotein; PA, Polymerase Acidic protein; PB1, Polymerase Basic protein 1; NLS, Nuclear Localisation Signal; ER, endoplasmic reticulum; TGN, trans Golgi network; PRR, Pattern Recognition Receptor; PAMPs, Pathogen Associated Molecular Patterns; DAMPs, Danger Associated Molecular Patterns; TLR, Toll-like Receptor; CLR, C-Type Lectin Receptor; RLR, Retinoic acid-Inducible gene-I (RIG-I)-like Receptor; NLR, Nucleotide Oligomerisation Domain (NOD)-like Receptor; ALR, Absent in Melanoma (AIM)-2 like Receptor; ASC, Apoptosis-associated Speck-like Protein containing a caspase recruitment domain (CARD); DC, Dendritic Cell; mTORC1, Mammalian Target of Rapomycin (mTOR) complex 1; ULK1, Unc-51-like autophagy activating kinase 1; PI3K, phosphatidylinositol 3-kinase; LC3, Microtubule-associated protein 1A/1B-light chain 3; LIR, LC3 interaction domain; MAVS, Mitochondrial Antiviral Signalling protein; NF-κB, Nuclear Factor-κB; IRF, Interferon Regulatory Factor; ROS, Reactive Oxygen Species; HSP, Heat Shock Protein; IPK, inhibitor of protein kinase; PKR, protein kinase R; eIF-2α, eukaryotic initiation factor 2α; BST-2, Bone marrow stromal antigen 2; ENaC, epithelial sodium channels; CFTR, cystic fibrosis transmembrane conductance regulator; PKC, protein kinase C; AnxA6, Annexin A6; ATP1B1, ATPase Na^+^/K^+^ Transporting Subunit Beta-1.

### 2.1. Influenza A Virus (IAV)

Influenza is a member of the *Orthomyxoviridae* family of viruses. There are four types of influenza virus, Influenza A-D. Influenza A virus (IAV) and Influenza B virus (IBV) are highly infectious in humans and are responsible for up to 1 billion seasonal flu infections each year [11,12]. Influenza is particularly severe in high risk populations, such as elderly or immunocompromised individuals, and is responsible for up to 650,000 deaths worldwide each year [13]. IAV, which causes the most severe respiratory illness, is further divided into subtypes based on the Haemagglutinin (HA) and Neuraminidase (NA) antigens expressed on the surface of the virus [11,14]. HA is responsible for binding to host sialic acid receptors for viral entry, while NA degrades the receptor and aids in viral egress [14]. IAV H1N1 and H3N2 are currently circulating in humans and these viruses are evolving rapidly, both by spontaneous mutation and gene segment reassortment [15]. Thus, Influenza viruses avoid detection, become resistant to antiviral treatments and suppress the immune response. It is well known that Influenza viruses avoid detection through antigenic changes in the envelope glycoproteins, and avoid natural immunity through the introduction of mutations in the Interferon (IFN)-antagonist Non-structural protein (NS)-1, the ion channel Matrix protein 2 (M2) and other proteins [16]. Furthermore, novel IAV strains continually emerge in birds and pigs which could potentially be introduced into humans resulting in an influenza pandemic. The rapidly evolving nature of Influenza limits the effectiveness of current vaccines and treatments, highlighting the need for further research into this virus and the identification of novel therapeutic strategies.

### 2.2. Influenza Proteins

The IAV and IBV genome is made up of 8 segments of negative sense single stranded RNA, all bound at each end by tetrameric RNA dependent RNA polymerase. Viral oligomeric nucleoprotein associates with the remaining viral RNA, forming a structure known as the viral ribonucleoprotein (vRNP) complex [17]. The gene segments each code for one or multiple proteins with the largest segments (segments 1–3) encoding the proteins of the RNA polymerase, Polymerase Acidic protein (PA), Polymerase Basic protein (PB)1 and PB2. The PB1 segment also encodes PB1-F2, which localises to the mitochondria and induces apoptosis [18]. Furthermore a truncated version of PB1, PB1-N40, can also be synthesized from the PB1 segment [19]. An alternate reading frame of PA encodes for PA-X [20] which suppresses host gene expression by targeting host RNA for degradation [21]. Two novel proteins which can be made for the PA segment, PA-N155 and PA-N185, have recently been identified, but their function is not yet known [22]. Segments 4–6, the next largest in size, encode the surface antigens, HA and NA, and also the nucleoprotein which has Nuclear Localisation Signals (NLSs) [23] allowing for translocation of vRNPs to the nucleus. Gene segment 7 encodes the Matrix Protein, M1, and through alternative splicing the Class IA viroporin, M2, while segment 8, the smallest segment, encodes the non-structural proteins, NS1 and NS2. NS1 interferes with the transcription of host mRNAs [24], including IFN-β, while NS2 mediates nuclear export of vRNPs [25]. M2 has an α-helical transmembrane domain which forms a tetramer complex through disulfide bonds [26]. M2 forms a proton channel which regulates PH at two important stages of the viral life cycle, during virus uncoating and viral maturation. M2 is present on the lipid envelope of the virus and causes virus acidification following endocytosis, which allows for dissociation of vRNPs from M1 on the inner surface the viral envelope [27]. M2 forms pores in the Golgi apparatus and maintains the pH in the lumen of the Golgi during transportation of HA from the endoplasmic reticulum (ER) to the cell surface in the trans Golgi network (TGN). M2 pumps H^+^ out of secretory organelles, maintaining a near-neutral pH, allowing for transport of HA to the cell surface in its native conformation [28,29,30]. The transmembrane domain of M2 has a conserved “HXXXW” motif, which is essential for ion channel gating, where Histidine 37 confers proton specificity and Tryptophan 41 ensures directionality of proton conductance [31,32]. Interestingly, M2 from Influenza IAV and M2 from Influenza IBV share very little sequence homology apart from this HXXXW motif [33].

### 2.3. Inflammasome Activation by M2

**Figure 2:**
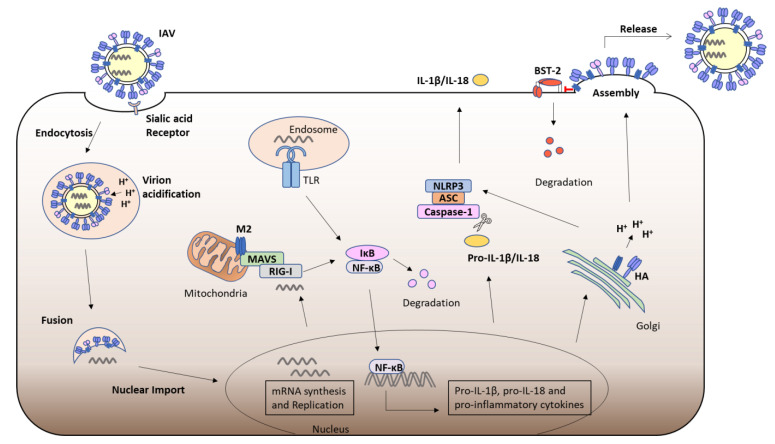
IAV M2 viral ion channel. The ion channel activity of M2 plays an important role in the IAV life cycle. IAV binds to sialic acid receptors on host target cells and is subsequently endocytosed. The ion channel activity of M2 allows for acidification of the virion causing dissociation of vRNPs. M2 then modifies the pH of the Golgi apparatus allowing for HA to be transported to the cell surface in its native conformation for virion assembly. Furthermore, M2 targets BST-2 for degradation thereby enhancing virion release. Several immune regulatory functions have also been attributed to M2. The presence of viral RNA in the cytoplasm or endosome triggers signaling cascades culminating in the proteasomal degradation of IκB proteins allowing for translocation of NF-κB to the nucleus and the upregulation of pro-inflammatory cytokines including pro-IL-1β and pro-IL-18. M2 can localise to the Mitochondria where it binds MAVS and promotes downstream NF-κB activation. The ion channel activity of M2 can also activate the NLRP3 inflammasome leading to the cleavage of pro-IL-1β and pro-IL-18 into mature IL-1β and IL-18, promoting inflammation and pyroptosis.

The cells of the innate immune system express a number of germlines encoded Pattern Recognition Receptors (PRRs), which sense invading pathogens by detecting Pathogen Associated Molecular Patterns (PAMPs), such as viral nucleic acid, or Danger Associated Molecular Patterns (DAMPs) and trigger an immune response [34]. PRRs include cell surface or endosomal Toll-like Receptors (TLRs) and C-Type Lectin Receptors (CLRs), as well as cytosolic Retinoic Acid Inducible (RIG-I)-like Receptors (RLRs), Nucleotide-binding and Oligomerisation Domain (NOD)-like Receptors (NLRs) and Absent in Melanoma (AIM)-2 like Receptors (ALRs) [34]. The presence of 5′ triphosphate viral RNA in the cytosol during Influenza infection triggers RIG-I activation; while TLR3 and TLR7 recognise the presence of influenza double stranded or single stranded RNA in the endosome, triggering signalling cascades that culminate in the upregulation of pro-IL-1β, pro-IL-18 and other pro-inflammatory cytokines [35]. NLRs and ALRs can also form inflammasomes, which are oligomeric complexes made up of the PRR, Apoptosis-associated Speck-like Protein containing a CARD (ASC) and Caspases. Their activation triggers the cleavage of pro-IL-1β and pro-IL-18, resulting in mature IL-1β and IL-18, which are then secreted from the cell and activate an inflammatory response. Inflammasome activation also induces the process of pro-inflammatory cell death, known as Pyroptosis [36]. Alternations in H^+^ homeostasis by the ion channel activity of M2 has been shown to trigger NLRP3 inflammasome activation in macrophages and dendritic cells (DCs), leading to IL-1β and IL-18 secretion, thus triggering an inflammatory response and contributing to viral pathogenesis [37] (Figure 2).

### 2.4. Autophagy and M2

Macroautophagy is an essential mechanism for protein degradation and recycling, whereby cellular components, such as aggregated proteins, damaged organelles or invading microbes, are surrounded by an autophagosome membrane which fuses with proteolytic lysosomes. Many viruses have developed mechanisms to promote or evade autophagy. Macroautophagy has been shown to promote influenza replication and reduce virus induced apoptosis [38,39]. Interestingly, M2 can stimulate the formation of autophagosomes, but it also inhibits the fusion of autophagosomes with lysosomes. Various signaling pathways can trigger autophagy in response to conditions such as ER stress or nutrient deprivation. Mammalian Target of Rapamycin complex 1 (mTORC1) is a negative regulator of the Unc-51-like autophagy activating kinase 1 (ULK1) complex, which activates the downstream class III phosphatidylinositol 3-kinase (PI3K) complex, leading to lipidation of microtubule-associated protein light chain 3 (LC3)-I to LC3-II and formation of the autophagosome membrane. M2 expression decreases AKT phosphorylation leading to decreased mTOR phosphorylation, triggering activation of autophagy in HEK293 cells [38]. IAV infection has also been shown to promote autophagy via the AKT-mTOR pathway in A549 cells [38,40]. IAV M2 has been shown to prevent autophagosomes from fusing with lysosomes, resulting in apoptosis of infected cells [41]. M2 interacts with Beclin-1, a member of the Class III PI3K complex and a key mediator of autophagosome formation and autophagosome-lysosome fusion. It was proposed that the accumulation of viral antigens within autophagosomes prevents their degradation for presentation via Major histocompatibility (MHC) molecules and thus subverts the activation of an adaptive immune response [41]. Interestingly, the ion channel activity of M2 is not required for inhibition of autophagosome-lysosome fusion. Furthermore, M2 has an LC3 interaction domain (LIR) within its C-terminal cytoplasmic tail which can promote LC3 redistribution to the plasma membrane, promoting filamentous budding and virion stability [42]. M2 can localise to the mitochondria where it interacts with the RLR adaptor protein Mitochondrial Antiviral Signaling protein (MAVS) leading to downstream Nuclear Factor (NF)-κB and Interferon Regulatory Factor (IRF)3 activation and the upregulation of their target genes [43] (Figure 2). Interestingly, the proton channel activity of M2 was found to trigger Reactive Oxygen Species (ROS) production which induced autophagy and enhanced MAVS signalling [43].

### 2.5. Effects of IAV M2

In addition to inflammasome activation, the influenza viroporin M2 has been linked to several other immune regulatory functions. A yeast 2 hybrid screen identified HSP40 as an interactor of IAV M2 [44]. HSP40 is known to regulate the 58kDa inhibitor of protein kinase (IPK), P58^IPK^, which inhibits PKR activity [45]. PKR is a serine-threonine kinase which is upregulated by IFN and plays an important role in the antiviral response to influenza. PKR is activated by dsRNA which induces its autophosphorylation, PKR then inhibits translation by phosphorylating the α subunit of eukaryotic initiation factor 2α (eIF-2α) [46]. P58^IPK^ is normally in a complex with HSP40 in an inactive state, but when activated can bind PKR and prevent its autophosphorylation. Following IAV infection, NP interacts with HSP40, leading to dissociation of P58^IPK^ and the inhibition of PKR [47]. During the early stages of viral replication, NS1 from IAV prevents the activation of PKR, allowing for the translation of viral proteins [48,49]. However, in the late stages of viral infection M2 interacts with HSP40 and P58^IPK^, stabilising the complex, resulting in PKR activation and increased cell death, which presumably aids viral release [44]. Bone marrow stromal antigen 2 (BST-2), also known as Tetherin, is another host factor upregulated in response to IFN, that inhibits the release of enveloped viruses from the cell. M2 has been shown to interact with BST-2 and promote its proteasomal degradation, enhancing the release of virus particles from infected cells [50]. (Figure 2) The cell cycle regulator, Cyclin D3, can inhibit viral assembly by competing with M1 for binding to M2 [51]. However, IAV can cause redistribution of Cyclin D3 from the nucleus to the cytoplasm where it is targeted for proteasomal degradation, contributing to cell cycle arrest which facilitates viral replication [51]. M2 has also been shown to downregulate the expression of two host ion channels, the amiloride-sensitive epithelial sodium channels (ENaC) and the cystic fibrosis transmembrane conductance regulator (CFTR) chloride channels. M2 enhances levels of ROS which activates protein kinase C (PKC), leading to endocytosis and subsequent proteasomal degradation of ENaC [52]. The ion channel activity of M2 also plays a role in the loss of CFTR, as this chloride channel is sensitive to alterations in pH; due to the alkaline pH of secretory organelles maintained by M2, CFTR is targeted for ubiquitination and degradation [53]. ENaC and CFTR are important regulators of secretion and absorption of fluid and electrolytes in the lung and their dysregulation by M2 can lead to pulmonary edema [54].

### 2.6. Targeting M2 Ion Channel Activity

Amantadine was first identified as a treatment for influenza in the 1960s, although its mechanism of action was not known at the time [55], it was later found to bind to the pore of the M2 ion channel [56]. However, the development of resistance to Amantadine and its derivative, Rimantadine, means that they are no longer an effective treatment for IAV, highlighting the need for novel therapeutic approaches. Annexins are a family of calcium and phospholipid binding proteins, that have emerged as potential targets for IAV therapy [57]. Annexin A6 (AnxA6), can interact with the cytoplasmic tail of M2 at the plasma membrane and inhibit virus budding [58]. AnxA6 can also cause sequestration of cholesterol in late endosomes, impairing IAV endosomal fusion and release of vRNP [59], and reduces the cholesterol availability for viral envelopes, thus impairing viral progeny [60]. M2 can also bind to Na^+^/K^+^-ATPase β1 subunit (ATP1B1), an interaction which is essential for viral replication [61]. The Na^+^/K^+^-ATPase pump inhibitors, cardiac glycosides, have been proposed as therapeutic targets for influenza virus infection [62].

## 3. HIV-1 Vpu

Abbreviations: HIV, Human Immunodeficiency Virus; AIDS, Acquired Immune Deficiency Syndrome; ART, anti-retroviral therapy; CD, Cluster of differentiation; CCR5, chemokine receptor 5; CXCR4, chemokine receptor 4; Vif, Viral infectivity factor; Vpr, Viral protein r; Nef, Negative factor; Vpu, Viral protein U; Vpx, Viral protein X; SIV, Simian Immunodeficiency Virus; β-TrCP, β-transducin repeat-containing protein; CK-II, Casein Kinase 2; SCF, Skp1-Cullin1-F-Box; ERAD, ER associated degradation; NMR, Nuclear magnetic resonance; TASK-1, two-pore acid sensitive potassium channel; ESCRT, Endosomal Sorting Complexes Required for Transport; AP2, adaptor protein complex 2; CCV, Clathrin Coated Vesicles; CAML, Calcium Modulating cyclophilin Ligand; AP1, adaptor protein complex 1; NTB-A, NK-T-and -B cell antigen; CCR7, C-C Chemokine type Receptor 7; NKT cells, Natural Killer T cells; TSPANS, Tetraspanins; AIDS-NHL, AIDS-related B-cell non-hodgkins lymphoma; ADCC, Antibody-dependent cellular cytotoxicity; IκB, Inhibitory-κB; ISG, Interferon Stimulated Gene; JAK, Janus Kinase; STAT, Signal transducer and Activator of Transcription; ISRE, Interferon Stimulated Response Element; Tsg, Tumour suppressor gene; UBP, Vpu binding protein; TPR, tetratricopeptide repeat; HMA, 5-(N,N-Hexamethylene) amiloride.

### 3.1. Human Immunodeficiency Virus (HIV)

Human Immunodeficiency Virus (HIV) is a retrovirus from the lentivirus genus. HIV is further divided into two main types, HIV type 1 (HIV-1) and HIV type 2 (HIV-2). HIV remains of global significance, with 37.9 million people infected with HIV worldwide in 2018 [63]. In the case of HIV-1, if left untreated, infection commonly leads to the development of Acquired Immune Deficiency Syndrome (AIDS). HIV-2 is considered less pathogenic, with many infected individuals remaining long-term non-progressors [64]. HIV-1 can be further divided into groups M, N, O and P, with group M being the most prevalent [65]. Although anti-retroviral therapy (ART) greatly improves the life expectancy of individuals with HIV [66], there remains no curative treatment, highlighting the need for novel therapeutic approaches. HIV-1 targets CD4 expressing cells of the immune system, mainly helper T cells. The glycoprotein gp120, on the surface of HIV-1 virions, binds to CD4 and a co-receptor chemokine receptor 5 (CCR5) or chemokine receptor 4 (CXCR4) on target cells [67]. This induces conformational changes in transmembrane gp41, exposing its N terminal fusion peptide which inserts into the host membrane. The transmembrane gp41 then folds into a hairpin structure, bringing the opposing membranes into close proximity and allowing for membrane fusion and the release of the viral capsid into the cytoplasm [68]. The conical capsid core protects the viral genome which is composed of two copies of positive sense single stranded RNA. Following reverse transcription, the proviral DNA is transported into the nucleus and becomes integrated into the host genome [69]. Proviral DNA is transcribed using host cellular machinery, making viral structural proteins (matrix, capsid, nucleocapsid, p6), envelope proteins (gp120 and gp41), enzymes (protease, reverse transcriptase and integrase), regulatory proteins (tat and rev) and the accessory proteins, Viral infectivity factor (Vif), Viral protein r (Vpr), Negative factor (Nef) and Viral protein U (Vpu) [70]. Interestingly, less infectious HIV-2 does not encode Vpu, but instead encodes Viral protein X (Vpx) [70]. Vpu is an 81 amino acid protein, with a single transmembrane domain which forms oligomers [71]. Vpu is a class IA viroporin and its ion channel activity is evolutionarily conserved from Simian Immunodeficiency Virus (SIV) to HIV, suggesting that this function is important for HIV-1 replication [72]. Vpu is expressed late in the viral life cycle and the two main functions of Vpu are the downregulation of cell surface CD4 and the enhancement of viral particle release.

### 3.2. Viral Protein u (Vpu) and CD4

**Figure 3:**
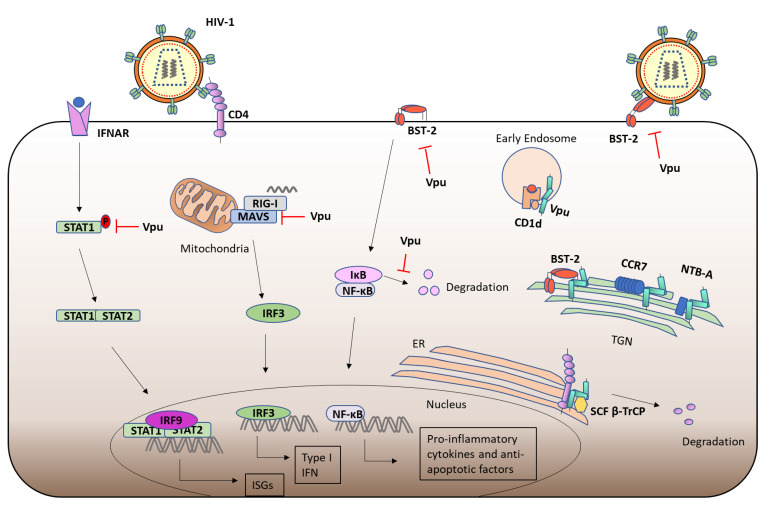
HIV-1 Vpu mediated immune evasion. Vpu downregulates the cell surface expression of various receptors, including the HIV-1 target receptor CD4, to evade immune responses and promote viral egress. Vpu sequesters CD4 in the ER, preventing its expression on the cell surface. Furthermore, Vpu recruits the SCF E3 ligase complex via the β-TrCP adaptor protein and promotes the ubiquitination and subsequent proteasomal degradation of CD4. Vpu sequesters BST-2, which inhibits HIV-1 viral egress by tethering virions to the cell surface, in the TGN and thereby promotes virion release. Similarly, Vpu sequesters other cell surface receptors including the chemokine receptor CCR7 and the NK cell receptor NTB-A in the TGN, thereby evading immune responses. Similarly, Vpu sequesters CD1d in early endosomes, further contributing to immune evasion. Vpu has also been shown to attenuate several innate immune signaling pathways. Vpu mediated downregulation of BST-2 attenuates NF-κB activation and the subsequent upregulation of pro-inflammatory cytokines and anti-apoptotic factors. Furthermore, Vpu binding to β-TrCP also prevents IκB degradation which is required for the nuclear localization of NF-κB and upregulation of target genes. Vpu downregulates MAVS, thus attenuating downstream IRF3 activation and subsequent antiviral Type I IFN induction. Furthermore, Vpu inhibits IFN induced STAT1 phosphorylation and downstream ISG expression.

Viral protein U (Vpu), from HIV-1 group M, downregulates the cell surface expression of a range of host proteins, including CD4 and BST2, to evade immune responses and promote HIV egress from infected cells [73]. Vpu is comprised of a short N-terminal domain, a hydrophobic TMD and a longer cytoplasmic domain, which includes the hinge region followed by two α-helical domains (H1 and H2) [73]. The cytoplasmic domain of Vpu encodes a highly conserved “DSGXXS” motif, which is required for interaction with the host protein β-transducin repeat-containing protein (β-TrCP). Casein Kinase 2 (CK-II) phosphorylates Vpu on specific serine residues (S52 and S56) within this motif, leading to the recruitment of the Skp1-Cullin1-F-Box (SCF) E3 ligase complex, through interaction with the F box protein β-TrCP substrate adaptor [74]. This functional E3 ligase complex can catalyse the poly-ubiquitination of target proteins such CD4. Vpu binding to β-TrCP causes its sequestration in the cytoplasm [75]. Vpu targeting of CD4 for SCF:β-TrCP-mediated poly-ubiquitination leads to subsequent recruitment of the ER associated degradation (ERAD) component VCP-UFD1L-NPL4 dislocase complex, which extracts CD4 from the ER to the cytosol for proteasomal degradation [76]. Furthermore, Vpu interacts with newly synthesised CD4, leading to its retention at the ER and its subsequent degradation [76] (Figure 3). The hinge region of Vpu has been shown to be required for efficient CD4 interaction and degradation [77]. Furthermore, a critical residue in the TMD of Vpu, W22, is also important for the downregulation of CD4 [78]. Loss of CD4 prevents the formation of CD4 complexes with the envelope precursor glycoprotein gp160 which facilitates proteolytic processing of gp160 into mature gp120 and gp41 [79]. Further to its role in downregulating CD4 expression, another important function of Vpu is to promote the release of HIV-1 virions from infected cells.

### 3.3. Vpu Ion Channel Activity

Notably, the TMD of Vpu has been shown to be important for promoting virion release from HIV-1 infected cells, while it is dispensable for CD4 degradation [80]. This finding led to the hypothesis that the ion channel function of Vpu may be important for promoting viral release. It was proposed that the modulation of cations across Vpu monomeric channels increases the permeability of host plasma membranes and thus promotes viral budding [81]. Vpu has been shown to enhance the membrane conductance of monovalent cations in amphibian oocytes, a process which was dependent on the Vpu TMD [82]. Although, this ion channel activity was later disputed and suggested to be an artifact of the overexpression of membrane proteins in oocytes and it was instead proposed that Vpu expression selectively inhibited the conductance of potassium ions in oocytes by the downregulation of cell surface proteins [83]. Nuclear magnetic resonance (NMR) analysis revealed that the hydrophobic α-helix in the TMD of Vpu transverses the membrane, while the two amphipathic α-helices of the C-terminal domain were parallel to the lipid bilayer [84]. The C-terminal domain promotes the oligomerisation of the TMD and stabilises conductance, while the TMD forms the ion channel pore when reconstituted in lipid bilayers in *E.coli* [85]. Vpu mimics the sequence of a mammalian two-pore acid sensitive potassium channel (TASK-1) and Vpu has been shown to interact with TASK-1 and inhibit its conductance [86]. TASK-1 conductance inhibits viral release, while Vpu oligomerisation with TASK-1 destabilises the transmembrane potential, removing this restrictive voltage barrier [87]. Furthermore, Vpu has also been shown to regulate potassium transport in *Saccharamyces cerevisiae* and induce membrane depolarization [88]. Membrane depolarization by Vpu likely aids viral egress while more research is required to determine the functional importance of the ion channel activity of Vpu.

### 3.4. Vpu and BST-2

The transmembrane IFN-α inducible antiviral factor, BST-2, retains newly budding HIV-1 virions at the cell membrane, leading to their subsequent endocytosis [89]; HIV-1 Vpu has been shown to downregulate the cell surface expression of BST-2 to evade this. HIV-1 Vpu has been shown to bind BST-2 and promote its SCF:β-TrCP mediated poly-ubiquitination and ERAD mediated proteasomal degradation [90], or endo-lysosomal degradation [91,92,93], thereby promoting the release of virions from the infected cell. Indeed, Vpu has been shown to promote the internalisation and lysosomal degradation of cell-surface BST-2 [94]. Furthermore, a component of the Endosomal Sorting Complexes Required for Transport (ESCRT), which is responsible for the sorting of ubiquitinated proteins for lysosomal degradation, has been shown to promote Vpu-mediated downregulation of BST-2 [93]. Importantly, Vpu can downregulate the cell surface expression of BST-2 in the absence of β-TrCP recruitment [95]. Vpu can modify the clathrin-dependent trafficking of BST-2, leading to its sequestration in endosomal compartments and preventing the expression of recycled BST-2 at the cell surface [96,97]. The recycling endosome has been further implicated in Vpu enhanced virion particle release [98], and serine-threonine ubiquitination of BST-2 by Vpu was hypothesised to modulate its endosomal trafficking and prevent recycling of BST-2 to the cell surface [99]. Furthermore, the endocytic clathrin adaptor protein complex 2 (AP2), has been shown to be required for optimal downregulation of BST-2 expression [92]. In the absence of Vpu, BST-2 is endocytosed and delivered to the TGN in a clathrin-dependent pathway [100]. Indeed, inhibition of the Clathrin-mediated endocytic pathway attenuates Vpu-mediated downregulation of BST-2 [96]. Vpu has recently been shown to interact with the V0 subunit C of Vacuolar ATPase (ATP6V0C), which was associated with expression of BST-2 in endosomal and lysosomal compartments [101]. Calcium Modulating cyclophilin Ligand (CAML) is a Vpu-interacting protein, that was suggested to play a role in Vpu mediated enhancement of viral egress, but was later found to have no effect on BST-2 expression or viral release in Cos-7 or HeLa cells [102,103]. The downregulation of BST-2 from the cell surface by the above mechanisms allows for viral egress.

Certain structural features of Vpu have been shown to be important for BST-2 inhibition. Vpu has been shown to bind BST-2 via interactions in their α-helical TMDs [104], and the sequence of the BST-2 TMD confers species specific targeting by Vpu [105,106]. A Study has also shown the importance of the N-terminus of Vpu for BST-2 antagonisation [107]. Furthermore, specific residues in the highly conserved hinge region of Vpu (E28 and L33), have also been shown to be important for BST-2 binding [77]. Critical residues in the hinge region (R30 and K31) allow for Vpu trafficking between late endosomes and the TGN, while the second α-helix of Vpu allows for accumulation of Vpu within the TGN, where it co-localises with BST-2 [108] (Figure 3). In the absence of the TMD, this C-terminal α-helix has also been shown to remove BST-2 from the site of virion assembly [109]. Furthermore, a C-terminal tryptophan (W76) has been shown to anchor Vpu in the lipid bilayer and enables the removal of BST-2 from virion assembly points [110]. The C-terminal domain of Vpu contains proximal tyrosine- and dileucine-based trafficking motifs, which are required for trafficking of Vpu and enhancement of viral release [97,111]. This canonical clathrin-sorting motif of Vpu allows for the recruitment of adaptor protein complex 1 (AP1), which is responsible for Vpu-mediated trafficking of BST-2 [112]. It has also been suggested that BST-2 inhibits the productive cell-to-cell transmission of Vpu-deficient HIV-1 in T cells [113], while other studies suggested that BST-2 promotes the formation of virological synapses between cells and that loss of Vpu promotes cell-to-cell transmission [114], possibly due to the increased concentration of virions tethered to cells. These studies further highlight the importance of Vpu mediated BST-2 antagonisation.

### 3.5. Immunomodulation by Vpu

Further to the downregulation of cell surface CD4, Vpu also modulates the expression of other cell surface receptors to evade the immune system and promote infection. Vpu downregulates the cell surface expression of the co-stimulatory molecule CD28, by promoting its lysosomal degradation. The S52 and S56 residue were shown to be essential for Vpu-mediated loss of CD28, indicating that Vpu utilises a similar mechanism to downregulate the cell surface expression of CD4 and CD28 [115]. The loss of CD28 alters T cell activation during HIV infection. In fact, the loss of CD28 on CD8+ T cells correlates with disease severity in HIV-1 infected patients [116]. Vpu has also been shown to downregulate the cell surface expression of the co-activation NK receptor NK-T-and -B cell antigen (NTB-A), causing its retention within the Golgi-compartment [117], thereby evading the NK mediated lysis of infected cells [118,119]. Similarly, Vpu has been shown to downregulate the cell surface expression of C-C Chemokine Receptor type 7 (CCR7), by sequestering CCR7 in the TGN [120]. Notably, the recruitment of SCF:β-TrCP is dispensable for Vpu-mediated downregulation of NTB-A and CCR7. Vpu also downregulates the cell surface expression of CD1d on DCs, inhibiting antigen presenting to Natural Killer T (NKT) cells [121], by sequestering CD1d in early endosomes and prevents its recycling to the plasma membrane [121] (Figure 3). Similarly Vpu downregulates the cell surface expression of the NK cell ligand CD155, thereby preventing the activation of NK cells and evading NK-mediated lysis of HIV-1 infected cells [122], in a mechanism requiring the TMD of Vpu [123].

Vpu has also been shown to be responsible for the downregulation of cell surface MHC class I molecules in HIV-1 infected cells [8], which would prevent the presentation of antigen to CD8+ T cells. Furthermore, a yeast two hybrid screen identified MHC class II invariant chain, CD74, as an interactor of Vpu and that Vpu mediated downregulation of mature MHC class II resulted in reduced antigen presentation from HIV-1 expressing monocytes [9]. Vpu also downregulates the surface expression of several Tetraspanins (TSPANs), including CD81 [124]. Vpu interacts with CD81 and downregulates its cell surface expression by lysosomal and proteasomal degradation in HIV-1 infected T cells [124], which may attenuate T cell activation [125]. Vpu also associates with the L-selectin (CD62L) leukocyte homing receptor, and sequesters it in perinuclear compartments, preventing the adhesion and signalling of HIV-1 infected CD4+ T cells [126]. Conversely, Vpu was shown to upregulate the expression of CD40 in endothelial cells, increasing cellular adhesion of B-lymphoma cells, which plays a role in the pathogenesis of AIDS-related B-cell non-hodgkins lymphoma (AIDS-NHL) [127].

Through the downregulation of cell surface receptors and co-stimulatory molecules, Vpu helps to create an environment favourable for viral replication. The loss of CD4 prevents repeat infection of the same cell, promotes maturation of virions and prevents Antibody-dependent cellular cytotoxicity (ADCC) [128], downregulation of BST-2 promotes virion release and loss of receptors, such as NTB-A, prevents immune detection and subsequent lysis of the infected cells and attenuates immune signaling.

In addition to its role sequestering virions within the cell, BST-2 can also trigger the canonical NF-κB pathway in response to viral infection [129]. NF-κB is bound by Inhibitory-κB (IκB) protein in unstimulated cells. Various signaling cascades culminate in the ubiquitination and degradation of IκB, leading to translocation of NF-κB to the nucleus, where it regulates gene expression (Figure 3). BST-2 induced NF-κB activation is attenuated by Vpu-mediated BST-2 degradation [129]. Furthermore, Vpu has also been shown to stabilise IκB, thereby preventing the nuclear translocation of NF-κB [130]. Nef triggers NF-κB activation early in HIV-1 infection to stimulate proviral DNA transcription, but Vpu, which is expressed later during replication, attenuates it [130]. βTrCP targets the IκB proteins for ubiquitination and subsequent proteasomal degradation. Vpu competes with other proteins for binding to βTrCP and as such attenuates IκB degradation and NF-κB activation [75,131]. This inhibition of NF-κB reduces the expression of anti-apoptotic factors leading to apoptosis in HIV-1 infected T cells [132]. More recently, RNA sequencing (RNA-seq) analysis has revealed that Vpu inhibits NF-κB regulated gene transcription, downregulating genes involved in the immune response (such as MHC class I), antiviral restriction factors (such as Interferon Stimulated Gene (ISG)15) and pro-inflammatory mediators [133]. In the absence of Vpu, PRRs of the innate immune system detect the presence of HIV-1, leading to IRF3 activation [134]. Vpu has been shown to inhibit Type I IFN production in response to HIV-1 viral infection by reducing the expression of MAVS in T cells [135]. Type I IFNs signal via the Janus Kinase (JAK)/Signal transducer and Activator of Transcription (STAT) signaling pathway, which upregulates the expression of ISGs. Vpu has also been shown to inhibit STAT1 phosphorylation in response to IFN-α, which prevents the binding of STAT1 to the Interferon Stimulated Response Element (ISRE) promoter site of target genes, thus attenuating the anti-viral effects of the JAK/STAT signalling pathway [136] (Figure 3). Through the downregulation of cell surface proteins and the regulation of the above signaling pathways, Vpu plays a role in the attenuation of both innate and adaptive immune responses.

### 3.6. Other Functions of Vpu

Further to its immunoregulatory functions, Vpu is also involved in the transport of virus-derived proteins. Expression of Vpu leads to the redistribution of viral capsid, gag, to the plasma membrane, where virion assembly occurs [137]. Furthermore, in the absence of Vpu, Gag and Env accumulate in clathrin-coated endosomal structures [138]. Following virion assembly, Vpu inhibits the endocytosis of budding virions from the cell surface in HeLa cells [139]. Similarly, Vpu prevents the Tumour suppressor gene (Tsg)101-mediated endocytosis of Gag from the cell surface [140]. Vpu-binding protein (UBP) was identified as a Vpu interacting cellular protein and Vpu inhibits the association of UBP with Gag [141]. UBP is a member of the tetratricopeptide repeat (TPR) family of proteins, which includes organelle-targeting proteins, and its overexpression restricts HIV-1 particle release in HeLa cells [141]. The above studies highlight the role of Vpu in virion assembly and release.

### 3.7. Targeting Vpu

The Vpu inhibitor 5-(N, N-Hexamethylene) amiloride (HMA) targets the ion channel activity of Vpu and was reported to inhibit HIV-1 replication in vitro [142]. Another amiloride analogue, N-[5-(1-methyl-1H-pyrazol-4-yl)-napthalene-2-carbonyl]-guanidine BIT225, was later found, which inhibited HIV-1 replication more effectively in Monocyte Derived Macrophages [143]. Furthermore, BIT225 inhibited HIV-1 replication in monocyte derived DCs and prevented the infection of co-cultured CD4+ T cells [144]. Moreover, BIT225 also reduced the viral burden in monocytes from patients with HIV-1 [145]. Interestingly, while this inhibitor targets the ion channel activity of Vpu, it does not affect Vpu- mediated downregulation of BST-2 [146].

## 4. HCV p7

Abbreviations: HCV, Hepatitis C Virus; DAA, Direct Acting Antiviral; RdRp, RNA dependent RNA polymerase; ORF, Open reading Frame; NTR, Non-translated Regions; DMVs, double membrane vesicles; MW, membranous web; SOCS, Suppressor of Cytokine Signalling; PBMC, Peripheral Blood Mononuclear cell; TRAF, tumour necrosis factor receptor associated factor; ERK, extracellular signal-related kinase; MAPK, mitogen-activated protein kinase; *N*N-DNJ, N-nonyl deoxynojirimycin.

### 4.1. Hepatitis C Virus (HCV)

Hepatitis C Virus (HCV) mainly infects hepatocytes and is the causative agent of hepatitis C liver disease, which is characterised by inflammation and fibrosis. Furthermore, HCV can lead to cirrhosis and hepatocellular carcinoma [147]. WHO estimated that there are currently 58 million people with chronic HCV infection worldwide. Direct Acting Antiviral (DAA) medicines are currently effective in treating 95% of cases [147]. HCV interferes with lipid metabolism and cholesterol homeostasis, as well as the immune response [148]. While acute HCV infection often remains asymptomatic, it is estimated that ~30% of individuals spontaneously clear HCV [147]. HCV is a small, enveloped, positive sense, single stranded, RNA virus from the *Hepacivirus* genus, within the *Flaviviridae* family and is subdivided into 7 genotypes and multiple subtypes [149], showing differential geographic distribution, progression and therapeutic responses. Currently, HCV genotype 1 is the most prevalent worldwide [150]. Due to high mutation rates caused by errors made by viral RNA dependent RNA polymerase (RdRp) during replication, HCV can also create a viral quasispecies within infected individuals [150]. HCV particles, ~50 nm in length, are made up of viral RNA encapsidated by core protein, surrounded by a host derived lipid bilayer envelope [151] in which the type I transmembrane envelope glycoproteins, E1 and E2, are anchored [152]. HCV circulates the blood in association with lipoprotein components [148]. E1 and E2 mediate cell entry in which receptor binding leads to clathrin-mediated endocytosis followed by pH-dependent fusion of the viral and endosomal membranes [151]. This facilitates the release of viral RNA (containing a single open reading frame (ORF), flanked by Non-translated Regions [NTRs]), into the cell [153]. This is translated into a single polyprotein, which is cleaved by host and viral proteases into ten mature structural and non-structural (NS) proteins, the core, E1, E2, p7, NS2, NS3, NS4A, NS4B, NS5A, and NS5B [154]. HCV and cellular proteins form a membranous web (MW), primarily consisting of double-membrane vesicles (DMVs) which originate from the ER, in which RNA replication takes place [155]. Following virus assembly, which occurs in close proximity to lipid droplets, the envelope is formed by budding at the ER and the mature lipoviral particle is thought to be released through the secretory pathway [153]. The first NS protein, p7, is a viroporin which is essential for virion assembly and release [156].

### 4.2. p7 Structure and Function

HCV p7 is a small protein, 63 amino acids in length, which oligomerises and forms cation selective channels [157]. HCV p7 normally localises to the ER, but can also be found in the mitochondria and at the plasma membrane [156,158]. HCV p7 is not required for cell entry, but is essential for viral assembly and release of infectious particles [156]. HCV p7 is a class IIA viroporin and the conductance of protons across p7 ion channels maintains pH in intracellular organelles, which contributes to the production and release of infectious particles [156,159]. Independent of its ion channel activity, p7 promotes the recruitment of the HCV core protein from lipid droplets to the ER for efficient viral assembly [160]. Furthermore, p7 is essential for capsid assembly and envelopment [161]. Incomplete cleavage of the HCV polypeptide results in precursor proteins, E2-p7-NS2 and E2-p7. Delayed cleavage of E2-p7 is important for mature viral particle assembly [162]. HCV p7 also causes depolarisation of the mitochondrial membrane and mitochondrial acidification which promotes HCV particle production [163]. The key features of the p7 structure are conserved among HCV genotypes. The structure of HCV genotype 1 p7 has been extensively studied and it comprises two TM regions, which are separated by a conserved dibasic loop domain [157]. NMR studies predict that HCV p7 monomers form helical hairpin-like structures, which are stable in the lipid bilayer and associate to form hexameric and heptomeric viroporins, with the N-terminal TM helix forming the channel pore [164]. Within the dibasic loop of p7 the K/R33 and R35 residues are highly conserved and are essential for p7 ion channel function and HCV viability [165,166]. R35 and N9 are essential for cationic specificity of p7 ion channels [167], while F25 has been proposed to function as a gating residue [164].

### 4.3. Immunomodulatory Functions of p7

**Figure 4:**
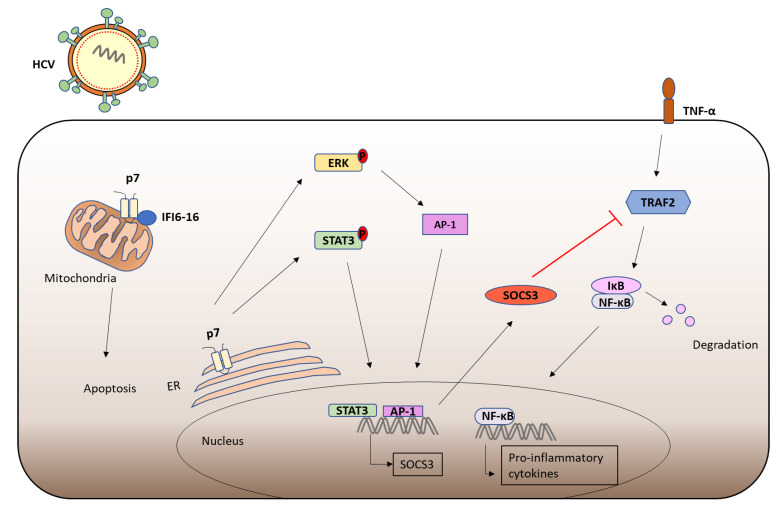
Immunomodulation by HCV p7. The viroporin p7 has recently been shown to contribute to immune evasion during HCV infection. p7, which localizes to the ER, has been shown to induce ERK and STAT3 phosphorylation which leads to subsequent SOCS3 upregulation. SOCS3 has been shown to attenuate TNF-α mediated NF-κB activation by targeting the signaling intermediate TRAF2. Thus, p7 inhibits the induction of NF-κB regulated genes, including pro-inflammatory cytokines. HCV p7 is also expressed at the mitochondria where it inhibits IFI6-16 leading to destabilization of the mitochondrial membrane and the induction of apoptosis.

Recently, immunomodulatory functions have been assigned to p7, which may contribute to the mild or even absent symptoms often observed during acute HCV infection. Higher levels of Suppressor of Cytokine Signaling (SOCS)3 were found in Peripheral Blood Mononuclear cells (PBMCs) from patients infected with HCV [168]. JAK/STAT signaling upregulates the expression of SOCS3, which targets JAK kinase and inhibits STAT3 activation, in a negative feedback loop [169]. SOCS3 also inhibits IL-1 mediated NF-κB activation by targeting the signaling intermediate tumour necrosis factor receptor associated factor (TRAF)6 and preventing its K63-linked ubiquitination [170]. Furthermore, SOCS3 has been shown to interact with TRAF2 and inhibit downstream NF-κB activation [168]. Recent findings showed that p7 expression stimulates extracellular signal-related kinase (ERK) MAPK and STAT3 phosphorylation leading to subsequent upregulation of SOCS3, and p7 expression inhibits TNF-α mediated NF-κB activation in hepatocytes [171]. Furthermore, the ion channel activity of p7 was shown to be required for the induction of SOCS3. HCV p7 has also been shown to interact with IFI6-16 and inhibit its antiviral activity via depolarization of the mitochondrial membrane [172]. IFI6-16 is rapidly induced in response to IFN and inhibits apoptosis through stabilisation of the mitochondrial membrane, where it is expressed [173]. These immunomodulatory functions of p7 may contribute to the mild pathology often associated with acute HCV infection (Figure 4).

### 4.4. Targeting p7

Given that p7 is essential for HCV infectivity in vivo [166], it is an attractive therapeutic target. Compounds which inhibit the oligomerisation of p7 were previously identified as potential therapies for HCV infection. Amantadine inhibits HCV p7 ion channel activity in vitro and was effective in some patients in combination with other HCV medications, with the variation in efficacy owing to differences in amino acid variation in p7 [174,175], where a mutation of L20 confers resistance to Adamantane [175,176]. The imino sugar derivative, N-nonyl deoxynojirimycin (NN-DNJ), inhibits p7 oligomerisation and subsequent ion channel formation [176]. However, mutations of F25 of p7, which are present in some HCV genotypes, confer resistance to *N*N-DNJ [176]. GSK-2, a p7 inhibitor developed by Glaxo-Smith-Kline, can inhibit HCV in a genotype-dependent manner [177]. HMA, which inhibits the ion channel activity of HIV-1 Vpu, has also been shown to block p7 ion channel conductance [159,178]. Moreover, BIT225 inhibits the ion channel activity of HCV p7 and reduced the viral load in patients with HCV [179].

## 5. HPV-16 E5

Abbreviations: HPV, Human Papillomaviruses; LCR, long control region; HSPG, heparin sulfate proteoglycans; EGFR, epidermal growth factor receptor; 16E5, HPV-16 E5; COX-2, cyclooxygenase-2; VEGF, vascular endothelial growth factor; PGE2, Prostaglandin E2 Receptor; cAMP, cyclic adenosine monophosphate; PKA, cAMP-dependent protein kinase; TRAIL, TNF-related apoptosis-inducing ligand; DISC, death-inducing signalling complex; KGFR, keratinocyte growth factor receptor; GM1, ganglioside-1; IRAK, Interleukin-1 receptor-associated kinase; TGF- β, Transforming Growth Factor-β; TGFβRII, TGF- β receptor II; HSV-1, Herpes Simplex Virus Type 1; HNSCC, head and neck squamous cell carcinoma.

### 5.1. Human Papillomavirus (HPV)

There are more than 100 Human Papillomaviruses (HPVs) which are very common worldwide and 14 of these are considered high-risk due to their ability to cause cancer [180]. Sexually transmitted high-risk HPV 16 and 18 are the main causative agents of cervical cancer [181]. Although the incidence of cervical cancer can be reduced by the administration of HPV vaccines [182], HPV infections are also linked to various other cancers including anal, penile and oropharyngeal [183]. HPVs belong to the *Papillomaviridae* family and have small, non-enveloped, icosahedral capsids. The genome of HPV 16 is circular, double stranded DNA (~7000–8000 base pairs), which contains three regions, the early (E) region, encoding the non-structural proteins (E1, 2 and 4–8), the late (L) region, encoding the structural proteins (L1 and L2 capsid proteins) and a long control region (LCR) [184]. HPV 16 targets basal epithelial cells, where L1 binds to heparin sulfate proteoglycans (HSPGs). This potentially activates the epidermal growth factor receptors (EGFR), which induces intracellular signaling cascades, such as the PI3K pathway and cyclophilin B activation, and leads to subsequent conformational changes in the capsid proteins [185]. This allows for L2 to bind to α6 integrins and subsequently Annexin A2 heterotetramers, a process which is potentially aided by tetraspanins, initiating endocytosis [185,186]. Early proteins E1 and E2 drive viral replication, while E6 and E7 are important for cell transformation. Newly synthesised virions are released from the terminally differentiated cells in the upper cornified layer and, therefore, the life cycle of HPVs is closely linked to cellular differentiation [187]. E5, 6 and 7 all encode viral oncoproteins. The oncoproteins E6, which targets the tumour suppressor p53 for degradation [188], and E7, which targets retinoblastoma (Rb) [189], are well-known to inhibit apoptosis and promote tumour development. While E5 is the least well characterised, it has been shown to form an ion channel, promote cell growth and play an important role in immune evasion.

### 5.2. HPV-16 E5

**Figure 5:**
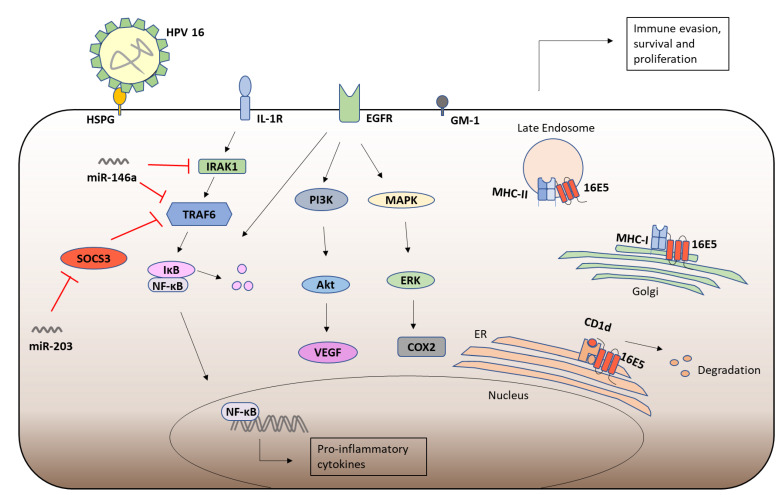
Immune modulation by HPV-16 E5. Following infection with HPV-16, which enters the cell via HSPG receptors, the ion channel E5 subverts the immune response. E5 sequesters CD1d in the ER, inhibits its trafficking to the cell membrane and promotes its proteasomal degradation. Similarly, 16E5 downregulates the cell surface expression of MHC class I and MHC class II molecules, thereby evading recognition by T cells. 16E5 upregulates the cell surface expression of GM1 which inhibits CD8+ T cell responses and contributes to EGFR activation. EGFR activated PI3K and MAPK signaling leads to COX-2 and VEGF upregulation, promoting cell survival and proliferation. Furthermore, 16E5 modulates miRNA expression. By repressing miR-203, 16E5 induces SOCS3 which inhibits NF-κB activation by targeting the signaling intermediate TRAF6. Furthermore, E5 enhances miR-146a which inhibits NF-κB activation by targeting signaling intermediates, IRAK1 and TRAF6. Thereby, E5 attenuates inflammation, subverts the activation of an adaptive immune response and promotes cell survival and proliferation.

HPV-16 E5 (16E5) forms hexameric ion channel structures and is sensitive to ion channel inhibitors [190]. The small (83 amino acid) hydrophobic 16E5 associates with internal membranes, including the ER, Golgi apparatus and the nuclear membrane [191]. 16E5 is a Class III viroporin with three TMDs and was predicted to have a luminal N-terminus and a cytosolic C-terminal domain, responsible for protein-protein interactions [192]. 16E5 has been shown to activate EGFRs and initiate downstream signaling pathways. Expression of 16E5 enhances ligand induced EGFR phosphorylation in HaCaTs [193] and keratinocytes [194]. 16E5 can modulate the endosomal pH [195] and delay their acidification, which was proposed to result in delayed degradation of EGFR after growth factor induced endocytosis, leading to enhanced recycling of EGFR to the cell surface in 16E5 expressing cells [196]. However, 16E5 has also been shown to inhibit the endosomal trafficking of EGF in a pH-independent manner [197], prevent the c-Cbl mediated ubiquitination and degradation of EGFR [198] and regulate the expression of cell surface lipid raft components which may enhance EGFR signaling [199]. 16E5 enhances MAPK and PI3K signaling, in response to EGF, thereby protecting the cell from apoptosis [200,201]. Enhanced EGFR signaling leads to the induction of cyclooxygenase-2 (COX-2) and vascular endothelial growth factor (VEGF) which promotes carcinogenesis [202,203] (Figure 5). 16E5 induced COX-2 promotes upregulation of the Prostaglandin E2 Receptor (PGE2), EP4, which also induces VEGF in cervical cancer cells [204].

16E5 inhibits apoptosis in cervical cancer cells by inducing the ubiquitination dependent degradation of the Bcl-2 family member, Bax, via a pathway dependent on COX-2, PGE2 and cyclic adenosine monophosphate (cAMP)-dependent protein kinase (PKA) [205]. Furthermore, E5 can inhibit TNF-related apoptosis-inducing ligand (TRAIL) mediated apoptosis, by perturbing death-inducing signaling complex (DISC) formation, and CD95L- triggered apoptosis, via the downregulation of CD95 and its cell surface expression [206,207]. Tumour protein p63 is a transcription factor expressed in basal epithelial layers and upon their differentiation p63 is downregulated by micro-RNA (miR)-203. Expression of 16E5 represses miR-203 and thus enhances p63 expression, thereby promoting cell proliferation [208]. 16E5 has also been shown to promote p63 expression in a miR-203-independent mechanism, via the downregulation of keratinocyte growth factor receptor (KGFR) expression. 16E5 alters the signaling and trafficking of KGFR which impairs keratinocyte differentiation [209] and inhibits autophagy [210]. Similarly, 16E5 reduces miR-196a in cervical cancer cells, which likely promotes proliferation and inhibits apoptosis [211]. Moreover, 16E5 expression alters genes involved in cell migration and promotes invasion of human cervical cancer cells [212,213]. 16E5 upregulates the growth factor receptor Met, via EGFR, which enhances motility in keratinocytes [214]. The N-terminal hydrophobic domain of 16E5 confers its ability to promote anchorage independent growth and keratinocyte invasion [215,216]. Thereby HPV16 E5 plays an essential role in cancer progression.

### 5.3. HPV-16 E5 and Immune Responses

HPV 16E5 also plays an important role subverting the immune response. 16E5 downregulates the cell surface expression of MHC class I molecules, by binding their heavy chain component and retaining them in the Golgi apparatus [217,218], reducing CD8+ T cells recognition of E5 expressing cells [219]. E5 has also been shown to alkalinise the Golgi apparatus by binding the 16kDa pore forming subunit of vacuolar-H^+^ ATPase (V-ATPase) [220], which may play a role in the retention of MHC class I molecules [219]. Interestingly, 16E5 mediated MHC class I downregulation is dependent on the di-leucine repeats in the N-terminal hydrophobic domain of E5 [218,221], which also allows for interaction with the MHC class I chaperone proteins, B-cell associated protein (Bap)31 [221,222] and Calnexin [223]. Similarly, 16E5 downregulates the cell surface expression of MHC class II molecules by preventing the degradation of invariant chain (Ii) chaperone protein in acidic endocytic compartments which is required for peptide loading [7]. Furthermore, the 16E5 induced upregulation of lipid raft component ganglioside-1 (GM1) at the cell surface inhibits CD8+ T cell activation [199]. E5 also inhibits the Calnexin dependent trafficking of CD1d, leading to its proteasomal degradation reduction in cell surface expression, thus preventing NKT cell recognition and CD1d mediated cytokine production [224]. By the above mechanisms, HPV16 subverts T cell and NKT cell responses (Figure 5).

HPV 16E5 also alters intracellular signaling pathways. miR-203 has been shown to target SOCS3 and therefore its downregulation by 16E5 promotes elevated levels of SOCS3 in 16E5 expressing cells, which may be a contributory mechanism used by HPV-16 to attenuate inflammation [208]. Similarly, expression of E5 has been shown to enhance levels of miR-146a [208], which inhibits NF-κB activation by targeting the signaling intermediates Interleukin-1 receptor-associated kinase (IRAK)1 and TRAF6 [225] (Figure 5). These findings help to explain the lack of an inflammatory response to HPV infection. Conversely, however, NF-κB plays an important role in E5 mediated COX-2 induction [202]. E5 has also been shown to downregulate the expression of the Transforming Growth Factor (TGF)-β receptor II (TGFβRII) and thus attenuates the TGF-β signaling pathway, promoting carcinogenesis [226]. Interestingly, E5 has been shown to promote IRF-1 mediated IFN-β expression [227], which promotes the clearance of viral episomes in cervical keratinocytes, leading to the emergence of HPV-16 integrants, an important step in cancer progression [228]. Conversely, 16E5 has also been shown to inhibit IFN-κ induction, attenuate subsequent ISG expression, and inhibit viral genome integration [229]. These studies highlight the intricate but broad roles of HPV 16E5 in disease progression, but also reveal the need for further investigation into the signaling cascades modulated by HPV-16 E5.

### 5.4. Targeting HPV-16 E5

Given that HPV-16 E5 is expressed in the early phase of the life cycle of HPV and given its immunosuppressive and oncogenic capabilities, it is a prime target for HPV-16 intervention and vaccination. Vaccination of mice with an adenovirus encoding E5 conferred some CD8+ T cell-mediated protection against tumour progression [230]. However, a 16E5 expressing recombinant vaccinia virus vaccine was unsuccessful at reducing tumour growth in rats [231]. Interestingly, a DNA vaccine encoding the HPV16 oncoproteins, E5, E6, and E7 genetically fused to Herpes simplex virus type 1 HSV-1 glycoprotein D conferred CD8+ T cell protective responses and anti-tumour effects in immunized mice [232]. E5 derived peptide-based vaccinations have also shown to be effective in preventing tumour growth in mice via the activation of specific CD8+ T cells [233,234]. Furthermore, a combination of E5 and E7 peptides from high risk HPVs was shown to be highly effective at preventing tumours in mice [235]. Similarly, mice immunized with an E5 DNA vaccine demonstrated anti-tumour effects and antigen specific T cell responses [236,237]. Specific epitopes from E5 that can stimulate T- and B-cell responses have been identified as potential therapeutics for HPV16 and as a vaccination target [238]. Interestingly, E5 confers resistance to PD-L1 immune checkpoint blockade therapy in patients with head and neck squamous cell carcinoma (HNSCC), via the downregulation of MHC, and Rimantadine has shown therapeutic benefit in patients [10]. Targeting 16E5 protein to DCs, in combination with PD-L1, has also shown therapeutic potential in a HPV16 related mouse tumour model [239]. HPV 16E5 targeting vaccines may allow for efficient immune clearance of HPV infected cells.

## 6. Severe Acute Respiratory Syndrome-Coronavirus (SARS-CoV) ORF3a

Abbreviations: SARS-CoV, Severe Acute Respiratory Syndrome-Coronavirus; PLpro, Papain-like protease; 3CLpro, 3C-like protease; nsp, non-structural protein; RTC, replicase-transcriptase complex; HMOX1, Heme oxygenase 1; PERK, PKR-like ER kinase.

### 6.1. Severe Acute Respiratory Syndrome-Coronavirus (SARS-CoV)-1

The outbreak of Severe Acute Respiratory Syndrome-Coronavirus (SARS-CoV)-1 in 2002 caused 8096 suspected cases and 774 deaths worldwide [240]. SARS-CoV-1 caused respiratory illness with symptoms including a cough, fever and shortness of breath and individuals with underlying health conditions, such as diabetes, were more at risk of developing severe respiratory disease [241]. SARS-CoV-1 is a member of the B lineage of betacoronaviruses and is very similar to SARS-CoV-2, the causative agent of the current pandemic [242]. There is a natural reservoir of these SARS-like CoVs in bats and SARS-CoV-1 was transmitted from bats to humans at an animal market in China, through intermediate species, including Himalayan Palm Civets [243,244]. Indeed SARS-CoV-2 likely has similar origins and was possibly transmitted to humans via intermediate species, such as Pangolins, at a Chinese animal market [245]. SARS-CoV-1 virus spread rapidly from human to human in droplets, but the virus also persisted in the air and on surfaces, leading to infection in the absence of close contact [246]. SARS-CoV-1 uses ACE2, which is expressed by epithelial cells in the respiratory tract, for cell entry [247]. SARS-CoV-1 is a single-stranded positive sense RNA virus. The viral particles are ~80 nm in length and the Spike (S) glycoproteins create crown (‘corona’ in Latin) like structures on the virion surface [248,249]. Following ACE2 binding by S protein, the virus is endocytosed and following membrane fusion the RNA genome is released into the cytoplasm [250]. The genome contains at least 14 open reading frames (ORFs), ORF1a/ORF1b, surface S glycoprotein, small envelope protein (E), outer membrane protein (M), nucleocapsid protein (N) and the accessory proteins ORF3a, 3b, 6, 7a, 7b, 8a, 8b, and 9b [251]. The large viral replicase polyproteins pp1a and pp1ab are translated from ORF1a and ORF1b and are cleaved by Papain-like protease (PLpro) and 3C-like protease (3CLpro) into 16 non-structural proteins (nsps), which form the membrane protected replicase-transcriptase complex (RTC) [252]. Following replication, N proteins encapsidate the RNA genome in the cytoplasm. At the same time, the M, S and E proteins are translated in the ER and are transported via the Golgi, where budding and particle formation occurs which are released by exocytosis in secretory vesicles [250]. There are three viral ion channels encoded by SARS-CoV-1, E, ORF3a, and ORF8a. The role of one of these ion channels, ORF3a, is discussed below.

### 6.2. ORF3a

ORF3a, 274 amino acids in length, is a class III viroporin with a large cytoplasmic domain. ORF3a forms a homotetrameric potassium sensitive ion channel in oocytes and it promotes viral egress from infected FRhK-4 cells [253]. ORF3a has been shown to localise to the plasma membrane, perinuclear regions and the Golgi apparatus [254,255,256] and affects Golgi fragmentation and accumulation of intracellular vesicles [257]. ORF3a has been shown to interact with Caveolin-1, by virtue of putative Caveolin binding sites [258]. The importance of Caveolin-1 binding is highlighted by the SARS-CoV-2 ORF3a mutant D155Y, which fails to bind Caveolin-1 affecting cell entry, membrane trafficking and virion assembly, while delaying viral egress and apoptosis [259]. SARS-CoV-1 ORF3a has been shown to induce apoptosis of infected Vero E6 cells, in a process dependent on Caspase 8 [260]. Similarly, ORF3a from SARS-CoV-2 has also been shown to promote apoptosis [261]. The potassium ion channel activity has been shown to be required for SARS-CoV-1 ORF3a induced apoptosis in Vero E6 cells [262]. ORF3a has been shown to interact with RIP3 which promotes ORF3a oligomerisation, causes lysosome damage, caspase 1 activation and necrotic cell death [263]. Interestingly, ORF3a induces fibrinogen in lung epithelial cells, which may contribute to the thrombocytopenia and elevated D-dimer levels observed in SARS-CoV-1 infected patients [264]. Similarly, ORF3a from SARS-CoV-2 has been shown to target Heme oxygenase (HMOX)1, which can inhibit platelet aggregation, thrombocytosis, and inflammation [265]. Furthermore, SARS-CoV-1 ORF3a has also been shown to be released from cells in membranous webs, potentially inducing apoptosis or other signalling cascades in surrounding cells [266].

### 6.3. ORF3a and the Immune Response

**Figure 6:**
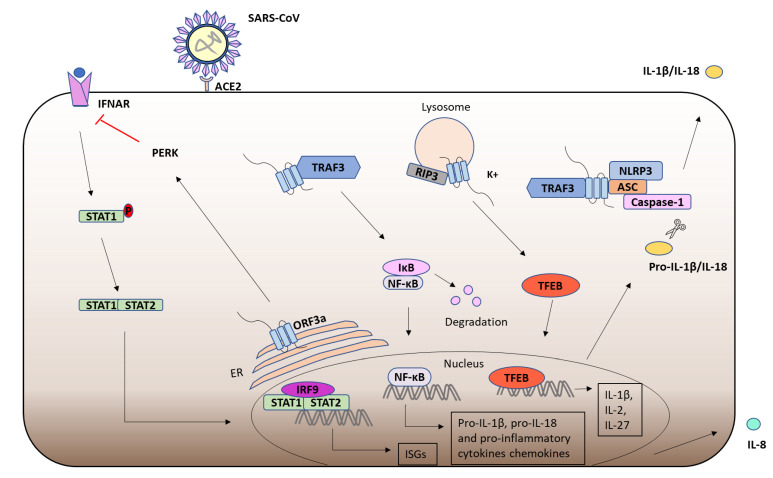
SARS-CoV ORF3a and the immune response. Following SARS-CoV infection of ACE2 expressing cells, ORF3a is expressed in the ER induces the PERK ER stress pathway resulting in the serine phosphorylation of IFNAR and attenuation of the IFN-α JAK/STAT signalling pathway and downstream ISG induction. ORF3a has been shown to co-localise with RIP3 in lysosomes and RIP3 has been shown to promote ORF3a oligomerisation leading to the activation of TFEB and subsequent pro-IL-1β, IL-2 and IL-27 induction. ORF3a has also been shown to interact with TRAF3 triggering downstream NF-κB activation and induction of pro-IL-1β, pro-IL-18 and IL-8. ORF3a has also been shown to promote inflammasome activation, through interaction with TRAF3 and via potassium efflux, leading to cleavage of pro-IL-1β and pro-IL-18. The release of IL-8, mature IL-1β and IL-18 from SARS-CoV promotes inflammation, contributing to the immunopathology associated with SARS-CoV infection.

SARS-CoV-1 suppresses anti-viral responses while promoting inflammation. ORF3a localises to the ER and has been shown to induce the PKR-like ER kinase (PERK) ER stress pathway, leading to serine phosphorylation of IFNAR1, triggering its ubiquitination and subsequent proteasomal degradation, thus attenuating JAK/STAT signalling [267] (Figure 6). SARS-CoV-1 ORF3a has also been shown to activate NF-κB and the NLRP3 inflammasome, resulting in the production of mature IL-1β [268]. In this study ORF3a was shown to induce the TRAF3 dependent ubiquitination of the NF-κB subunit p105 and ASC in a process independent of ORF3a ion channel activity [268]. Potassium efflux by ORF3a has also been proposed to activate the NLRP3 inflammasome [263] (Figure 6). Furthermore, the ORF3a mediated upregulation of NF-κB and JNK enhances the expression of the chemokine IL-8 in lung epithelial cells, which likely contributes to the pathological inflammation associated with SARS-CoV-1 [269]. Furthermore, ORF3a has also been shown to promote the nuclear localisation of Transcription Factor EB (TFEB), which can upregulate the transcription of cytokines such as IL-1β, IL-2 and IL-27 [263]. Interestingly, ORF3a from SARS-CoV-2, but not SARS-CoV-1, can block autophagosome fusion with lysosomes, thus protecting the virus from destruction [270]. Furthermore, ORF3a from SARS-CoV-2 has been shown to bind TRIM59, an E3 ligase which regulates NF-κB and IRF activation [271]. Moreover, expression of SARS-CoV-2 ORF3a in *Drosophila* induced apoptosis and inflammation in the central nervous system, suggesting that SARS-CoV-2 ORF3a might contribute towards the symptoms of post-COVID syndrome [272].

## 7. Polyomavirus Agnoprotein

Abbreviations: BKV, BK polyomavirus; JCV, polyomavirus JC; α-SNAP, α-soluble N-ethylmaleimide sensitive fusion attachment protein; PCNA, Proliferating cell nuclear antigen; PML, progressive multifocal encephalopathy; GMCSF, granulocyte-macrophage colony-stimulating factor.

### 7.1. Polyomavirus Agnoprotein

Another viroporin of interest, agnoprotein, is expressed by a subset of human polyomaviruses, including BK polyomavirus (BKV) and polyomavirus JC (JCV) and the closely related to simian SV40 [273]. BKV and JCV cause asymptomatic infection in healthy individuals but can cause severe disease in immunocompromised individuals. Polyomaviruses are small unenveloped double stranded DNA viruses with icosahedral capsids [273]. Agnoprotein, encoded by the late coding region of polyomaviruses, is a small hydrophobic phosphoprotein which is predominantly expressed in the cytoplasm but accumulates in the perinuclear regions and can also be found in the nucleus [274]. The NMR structure of JCV agnoprotein revealed that it possesses a major α-helix which is essential for oligomerisation, a minor α-helix domain, and intrinsically unstructured regions which likely facilitate interaction with diverse partners [275,276]. Agnoproteins share high sequence similarity in the N terminal and central domains but have diverse C terminal regions [273].

### 7.2. BKV Agnoprotein

Interestingly, Agnoprotein from BKV has been shown to co-localise with lipid droplets [277]. BKV agnoprotein has been shown to promote nuclear release and viral egress via interaction with the vesicular trafficking protein α-soluble N-ethylmaleimide sensitive fusion (NSF) attachment protein (α-SNAP) [278]. Proliferating cell nuclear antigen (PCNA) is another interacting partner of BKV agnoprotein which inhibits viral DNA replication during the late stages of infection when virion assembly occurs [279]. BKV Agnoprotein of BKV has also been shown to localise to the mitochondria during the late phase of viral replication where it disrupts the membrane potential and induces mitophagy, the degradation of the mitochondria by autophagy [280]. This attenuates MAVS and STING signalling pathways preventing downstream IRF3 activation and subsequent Type I IFN induction, thus evading the innate immune response and enhancing BKV replication [280].

### 7.3. JCV Agnoprotein

JCV agnoprotein has been shown to localise to the ER during early infection and the plasma membrane during the late stages of infection, increase membrane permeability to Ca^2+^ and promote virion release [281]. JCV agnoprotein interacts with viral capsid protein 1 (VP1) and plays an important role in capsid formation and virion assembly [282]. Furthermore, it has been shown to interact with JCV large T-antigen and promotes its DNA binding activity which likely leads to increased viral DNA replication [283]. The N-terminal of JCV agnoprotein has been shown to interact with p53 which disrupts cell cycle progression [284]. Furthermore, JCV agnoprotein interacts with Ku70 leading to re-localisation of Ku70 to the perinuclear regions and inhibition of DNA repair [285]. JCV Agnoprotein has also been shown to inhibit CG-4 rat oligodendrocyte cell differentiation and promote apoptosis [286]. Agnoprotein is expressed predominantly in the cytoplasm of JCV infected oligodendrocytes during progressive multifocal encephalopathy (PML), a demyelinating disease of the central nervous system caused by JCV infection [287]. Agnoprotein from JCV polyomavirus has been shown to impair CXCL5 chemokine release from CG4 oligodendrocytes causing dysregulation of glycogen synthase kinase (GSK3) and MAPK signalling pathways leading to neuronal apoptosis [288]. This likely contributes to neuronal/axonal injury in the pathogenesis of PML [288]. Interestingly, agnoprotein can be released from JCV infected oligodendrocytes and internalised by neighbouring cells which inhibits granulocyte-macrophage colony-stimulating factor (GM-CSF) transcription in Glial cells and impairs monocyte maturation, phagocytic activity and migration towards activated astrocytes [289,290]. These findings may help to explain the lack of inflammation during PML progression. Mutational NMR studies have revealed a specific region of the α-helical domain (the L29, L32 and L36 region) as a potential target for small molecule inhibition which would prevent agnoprotein dimerisation for the treatment of JCV infection and subsequent PML [273].

## 8. Concluding Remarks

The recent COVID-19 pandemic has highlighted the potential benefit of an in-depth knowledge of infectious viruses and the mechanisms utilised by viruses to modulate immune responses. While vaccines are an effective prophylactic treatment for many viruses, the need for successful therapeutics remains due to factors such as the potential emergence of novel strains and vaccine hesitancy. Viral ion channels have varying roles in the viral life cycle and have diverse immunomodulatory effects. Given their immunoregulatory capabilities, they are a prime target for the treatment of infectious viruses. This review shows that viral ion channels have many functions and their ability to form structural channels within host membranes adds an additional level of pathogen modulation that needs to be explored further. Indeed, future investigations into the role of viroporins will likely uncover novel therapeutic targets for the much-needed treatment of existing and future emergent viruses.
